# Predictivity of the comorbidity indices for geriatric syndromes

**DOI:** 10.1186/s12877-022-03066-8

**Published:** 2022-05-19

**Authors:** Kubra Canaslan, Esra Ates Bulut, Suleyman Emre Kocyigit, Ali Ekrem Aydin, Ahmet Turan Isik

**Affiliations:** 1 Department of Internal Medicine, Sinop Turkeli State Hospital, Sinop, Turkey; 2 Department of Geriatric Medicine, Adana City Training and Research Hospital, Adana, Turkey; 3 Department of Geriatric Medicine, University of Health Sciences, Tepecik Training and Research Hospital, Izmir, Turkey; 4 Department of Geriatric Medicine, Sivas Numune Hospital, Sivas, Turkey; 5 Department of Geriatric Medicine, Faculty of Medicine, Dokuz Eylul University, Izmir, Turkey; 6 Yaşlanan Beyin Ve Demans Unitesi, Geriatri Bilim Dalı Dokuz Eylul Universitesi Tıp Fakultesi, Balcova, 35340 Izmir, Turkey

**Keywords:** Comorbidity indices, Comprehensive geriatric assessment, Geriatric syndromes, Older adults

## Abstract

**Background:**

The aging population and increasing chronic diseases make a tremendous burden on the health care system. The study evaluated the relationship between comorbidity indices and common geriatric syndromes.

**Methods:**

A total of 366 patients who were hospitalized in a university geriatric inpatient service were included in the study. Sociodemographic characteristics, laboratory findings, and comprehensive geriatric assessment(CGA) parameters were recorded. Malnutrition, urinary incontinence, frailty, polypharmacy, falls, orthostatic hypotension, depression, and cognitive performance were evaluated. Comorbidities were ranked using the Charlson Comorbidity Index(CCI), Elixhauser Comorbidity Index(ECM), Geriatric Index of Comorbidity(GIC), and Medicine Comorbidity Index(MCI). Because, the CCI is a valid and reliable tool used in different clinical settings and diseases, patients with CCI score higher than four was accepted as multimorbid. Additionally, the relationship between geriatric syndromes and comorbidity indices was assessed with regression analysis.

**Results:**

Patients’ mean age was 76.2 ± 7.25 years(67.8% female). The age and sex of multimorbid patients according to the CCI were not different compared to others. The multimorbid group had a higher rate of dementia and polypharmacy among geriatric syndromes. All four indices were associated with frailty and polypharmacy(*p* < 0.05). CCI and ECM scores were related to dementia, polypharmacy, and frailty. Moreover, CCI was also associated with separately slow walking speed and low muscle strength. On the other hand, unlike CCI, ECM was associated with malnutrition.

**Conclusions:**

In the study comparing the four comorbidity indices, it is revealed that none of the indices is sufficient to use alone in geriatric practice. New indices should be developed considering the complexity of the geriatric cases and the limitations of the existing indices.

## Background

The world's population is getting older, and one of every ten people is over 65 years of age in 2021 [1], while it is expected to increase to one of every six people in 2050. [2] Accordingly, chronic diseases and comorbidities increase with aging. Therefore, older adults who already have a chronic disease should be evaluated for another chronic condition [3]. In literature, the number of newly diagnosed diseases in a year over 80 years old is five times higher than between 0–20. [4] Another study showed that men and women over 80 years old have an average of 3.24 and 3.57 chronic diseases, respectively [4]. In a study designed by our research group involving 1579 older adults, the average number of comorbidities was reported to be 4.36. [5]

Due to multiple comorbidities, overlapped signs and symptoms may cause difficulty in diagnosis [3]. Moreover, the co-existence of polypharmacy and multi-morbidity has become a significant problem due to inappropriate medications and adverse drug reactions. [6] These all may lead to misdiagnosis, delay in diagnosis, increase in disease burden [7, 8] and are associated with a high admission rate to health services, prolonged hospitalization, increased healthcare costs, and even mortality [4, 5, 7, 9, 10].

The functional, cognitive, psychosocial, and nutritional status of older adults should guide the treatment decision. CGA, which allows multidimensional holistic assessment of older adults, should predict the benefits and side effects of treatment and outcomes of the interventions on older people [11]. Furthermore, assessment of comorbidities constitutes an essential part of CGA [12]. Evaluation of patients with CGA may not be possible in daily practice because of time constraints. Potential time-saving, objective, and user-friendly screening tools or indices are needed to improve health care quality in older patients. In this context, comorbidity indices may be beneficial and practical to define complex patients. Comorbidity indices are used to evaluate clinical prognosis and comorbidity adjustment objectively. [7] A qualified comorbidity index should represent the patient's chronic diseases and severity with a single score enabling estimation outcomes of the patients. [13] An ideal comorbidity index is also expected to reflect geriatric syndromes, such as frailty, and an older patient's physical and functional status, which are closely related to clinical outcomes. [11] At this point, existing indices may not provide a good illustration of patients' general health status. A study comparing six different indices was pointed out that none of the indices were as valuable as CGA parameters in predicting 5-year mortality. [14]

Therefore, this study aims to determine an appropriate comorbidity index for geriatric patients and compare four comorbidity indices, which are most frequently used in older adults, to predict geriatric syndromes.

## Methods

### Sample size

This study was designed as retrospective and cross-sectional. A total of 2935 patients admitted and hospitalized in a university hospital geriatrics inpatient clinic between January 2015 and February 2019 were screened retrospectively. Three hundred sixty-six patients whose hospital records were suitable were included in the study.

**Fig. 1:**
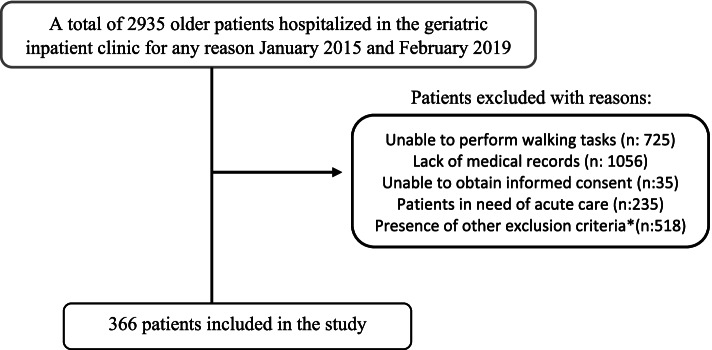
The flowchart of the study. *Patients who were transferred to another clinic, died during the follow-up, refused to complete the treatment, and were hospitalized for only intravenous drug infusion or transfusion

All patients over 60 years without exclusion criteria were included in the study. Patients were excluded if they transferred to another clinic, died during the follow-up, refused to complete the treatment, and were hospitalized for only intravenous drug infusion or transfusion. Patients who needed acute care, including acute coronary syndrome, gastrointestinal bleeding, acute cerebrovascular event, sepsis, respiratory distress, and unable to complete CGA parameters (unable to walk and hear) were also excluded. The flowchart of the study is shown in Fig. 1.

### Demographic features, CGA parameters, and geriatric syndromes

Sociodemographic features (sex, age, educational status), length of stay (LOS), and medications were reviewed. As a part of CGA, Montreal Cognitive Assessment Scale (MOCA)(for patients with nine years or more education) [15], Mini-Mental Status Examination (MMSE) [16] (for patients with 5–8 years of education) for neurocognitive evaluation; Yesavage Geriatric Depression Scale (YGDS) [17] for mood assessment; Lawton-Brody Instrumental Activities of Daily Living (IADL) [18] and Barthel Index for basic activities of daily living (BADL)[19] for functionality; Tinetti Performance and Mobility Assessment (POMA) [20] for assessment of balance and gait; Mini Nutritional Assessment short-form(MNA-SF) [21] for nutritional evaluation were applied [5]. MoCA scores were converted to MMSE[22]. Eight geriatric syndromes were evaluated in the patients.

Depression and Dementia were diagnosed according to the Diagnostic and Statistical Manual of Mental Disorders, Fifth Edition (DSM-V) criteria.

Orthostatic Hypotension: Postural blood pressure changes were evaluated via the Head-Up Tilt Table Test (HUT). Orthostatic hypotension is considered of fall in systolic blood pressure > 20 mmHg and/or diastolic blood pressure > 10 mmHg within the first 3 min after standing up from the supine position. [23]

Frailty: Frailty was defined using the Fried Physical Frailty Scale. [24] Walking speed and handgrip strength were measured using the 4-m walking test and JAMAR hydraulic hand dynamometer (Model J00105, Lafayette, USA). Patients were accepted as frail in the presence of three or more of the following five criteria: exhaustion, weight loss, weakness, slow walking speed, and low physical activity. Additionally, the timed up and go was measured as the overall time to complete the patient standing from a chair, walking 3 m, and returning to sit down. [25]

Polypharmacy was defined as the concurrent use of 5 and more drugs [5].

Urinary incontinence was diagnosed if the patient experienced involuntary urinary loss in the past three months except for urinary tract infection [26].

Fall was defined as an event that resulted in a person coming to rest inadvertently on the ground or floor or other lower level in the last year. [26]

Malnutrition: MNA-SF classified Nutritional status as malnourished when the score ≤ 7. [27]

### Comorbidity indices

Comorbidities are assessed with four different indices; Charlson Comorbidity Index (CCI), Elixhauser Comorbidity Measure (ECM), Geriatric Index of Comorbidity (GIC), and Medicine Comorbidity Index (MCI). CCI score was calculated based on the International Classification of Diseases (ICD) diagnosis codes with 19 categories weights from 1 to 6. Metastatic solid tumor and HIV has the highest points (6 points). ECM categorizes patients according to the burden of 30 comorbidities. GIC classifies patients for 15 chronic conditions considering the presence and severity of the diseases. Each condition is grouped into four classes (0 = absence of disease, 1 = asymptomatic disease, 2 = symptomatic disease requiring medication but under satisfactory control, 3 = symptomatic disease uncontrolled by therapy, and 4 = life-threatening or the most severe form of the disease). MCI was designed to evaluate 20 chronic diseases and the drugs used concerning these diseases. Patients were scored with CCI between 0 and 37 [28], ECM between -19 and 89 [29], MCI between 0–28, [30], while GIC [11] was classified as patients 0 to 4. Firstly, according to the CCI score, patients were divided into two groups to define the multimorbid group. (Low CCI: CCI score 0–4, high CCI: 5 and higher). Then, CCI, ECM, and GIC are grouped according to the score. (CCI group 1:0, group 2: 1–2, group 3:3–4, group 4: 5 and more; ECM group 1: below 0, group 2: 0, group 3: 1–4, group 4: 5 and more; GIC group1:1, group2:2, group3:3, group4:4).

### Statistical analysis

Frequency and percentages were used for categorical variables, and mean ± standard deviation was used for continuous variables in Table 1 for demographic features, laboratory data, comorbidities, CGA parameters, and comorbidity scores. Normal distribution for continuous variables was assessed using the Kolmogorov–Smirnov test. Categorical variables were expressed as percentages (%), and continuous measures were shown as mean ± standard. The first quartile (Q1) and the third quartile (Q3) of the non-normal distributed data were shown. The association of geriatric syndromes and the four index scores were shown using logistic regression analysis. Multinomial logistic regression analyses were used to determine the relationship between the aforementioned ECM, GIC, and CCI subgroups and CGA parameters adjusted for age and gender. The odds ratio (OR) was measured with a 95% confidence interval (CI), and group 1 was admitted as the reference group. A p-value of less than 0.05 was considered statistically significant. SPSS 22.0 (SPSS Inc.) was used for all statistical analyses.

**Table 1:** Demographics, comprehensive geriatric assessment parameters, and laboratory measurements of the patients according to multimorbidity

Variables	**Low CCI** **(Multimorbidity – group)** **(*n*= 311)**^a^	**High CCI** **(Multimorbidity + group)** **(*n*= 55)**^a^	***p*** value^*^
*DEMOGRAPHICS*			
Age	77 [71–81]^a^	77 [71–84]^a^	0.13
Sex (Female %)	66.8	74.5	0.27
Education (> 8 years %)	30.9	29.4	0.17
Number of Drugs	5 [3–8]^a^	8 [7–11]^a^	< 0.01^*^
Length of Stay	5 [4–7]^a^	6 [5–7]^a^	0.01^*^
*GERIATRIC SYNDROMES (%)*			
Urinary incontinence	54.0	50.9	0.77
Falls	42.4	43.6	0.88
Dementia	21.9	43.6	< 0.01^*^
Malnutrition	6.3	11.1	0.24
Depression	42.1	47.3	0.55
Frailty	44.2	54.3	0.26
Polypharmacy	60.8	92.7	< 0.01^*^
Orthostatic Hypotension	18.6	19.1	0.93
*COMORBIDITIES (%)*			
Hypertension	71.1	92.7	< 0.01^*^
Coronary Artery Disease	18.0	34.5	0.03^*^
Congestive Heart Failure	2.9	9.1	0.44
COPD	6.8	33.6	< 0.01^*^
Cerebrovascular Disease	7.4	21.8	< 0.01^*^
Diabetes Mellitus	29.3	65.5	< 0.01^*^
Hyperlipidemia	65.3	56.4	0.20
*COMORBIDITY INDICES*			
Charlson Comorbidity Index	2 [1–3]^a^	5 [5, 6]^a^	< 0.01^*^
Elixhauser Comorbidity Measure	2 [(-2)-7]^a^	9 [4.75–13.25]^a^	< 0.01^*^
Geriatric Index of Comorbidity	2 [2–2]^a^	2 [2, 3]^a^	0.02^*^
Medicines Comorbidity Index	3 [2–4]^a^	5 [4–7]^a^	< 0.01^*^
*COMPREHENSIVE GERIATRIC ASSESSMENT PARAMETERS*			
MMSE	25 [19–29]^a^	26 [18.75–29]^a^	0.61
YGDS	3 [1–6]^a^	2 [0–6]^a^	0.41
POMA_T_	26 [21–28]^a^	24 [18–27]^a^	0.10
MNA	13 [11–14]^a^	13 [10–13.25]^a^	0.33
BADL	90 [80–95]^a^	90 [60–95]^a^	0.03^*^
IADL	16 [9–22]^a^	14 [6–20]^a^	0.08
Timed Up & Go Test	14 [11–20]^a^	13.75 [10.72–20]^a^	0.94
*LABORATORY VALUES*			
Hemoglobin (g/dL)	12.40 [11.50–13.42]^a^	11.60 [10.70–12.60]^a^	< 0.01^*^
eGFR (mL/min/1.73 m2)	80.63 [65.08–89]^a^	50.20 [42.30–59.78]^a^	< 0.01^*^
LDL (mg/dL)	129 [106–154]^a^	116 [86–138]^a^	0.01^*^
Albumin (g/dL)	4.11 ± 0.72	3.91 ± 0.41	0.01^*^
Folic acid (ng/mL)	6.70 [5.43–9.57]^a^	6.62 [4.55–9]^a^	0.30
Vitamin B12 (pg/mL)	333 [216.25–469.75]^a^	351 [207–536]^a^	0.52
25-OH D vitamin (ng/mL)	19.60 [13.03–24.86]^a^	19.11 [15.34–26.62]^a^	0.45

*25 (OH)-D* 25-Hydroxy Vitamin D, *BADL* Basic Activities of Daily Living (0–100), *IADL* Instrumental Activities of Daily Living (0–23), *BMI* Body Mass Index, *eGFR* estimated Glomerular Filtration Rate, *COPD* Chronic Obstructive Pulmonary Disease, *LOS* Length of stay, *MMSE* Mini-Mental State Examination (0–30), *MNA-SF* Mini-Nutritional Assessment-Short Form (0–14), *OH* Orthostatic Hypotension, *POMA*_T_ Performance-oriented Mobility Assessment-Total (0–28), *TSH* Thyroid Stimulant Hormone, *YGDS* Yesevage Geriatric Depression Scale (0–15)

^a^The first quartile (Q1) and the third quartile (Q3) of the non-normal distributed data were shown in the parenthesis

^*^Bold p values indicate statistical significance *p* < 0.05

## Results

Of the 366 patients, 249 (67.8%) were female, and the mean age was 76.2 ± 7.25 years. The most common comorbidities were hypertension (74.3%), diabetes mellitus (34.7%), and dementia (25.1%). When patients were divided into two subgroups according to their CCI score, there 55 (15.1%) patients were in the high CCI group. No significant differences between the two groups were revealed in terms of age, gender, and education level. Patients in the high CCI group were using more drugs, and their length of stay in hospital was more extended than low CCI group (p < 0.05). The rates of dementia and polypharmacy were significantly higher in the multimorbid group (p < 0.001), while the rate of other common geriatric syndromes was similar between groups. As expected, the frequency of chronic diseases was higher in the multimorbid group, except for congestive heart failure and hyperlipidemia. When the comorbidity scores were calculated, the mean CCI score, ECM score, GIC score, and MCI score were higher in the high CCI group (p < 0.01). BADL scores were worse in the high CCI groups, while other CGA parameters showed no significant difference. Patients in the low CCI group have higher levels of hemoglobin, glomerular filtration rate (eGFR), LDL, albumin (p < 0.05). Demographic characteristics, laboratory results, and CGA parameters of patients are shown in Table 1.

When the association of indices with common geriatric syndromes was reviewed, all four indices were linked with frailty and polypharmacy(p < 0.05). Only ECM was associated with malnutrition and depression (p < 0.05). However, there was an inverse relationship between ECM score and depression. Low muscle strength and low walking speed, which are also the components of sarcopenia, were related to CCI score, while falls were only associated with GIC (p: 0.04). When age and sex were adjusted, all relationships were preserved except frailty with GIC score (Table 2).

**Table 2:** Association of the comorbidity indices with common geriatric syndromes

	**CHARLSON COMORBIDITY INDEX**		**MEDICINES COMORBIDITY INDEX**		**GERIATRIC INDEX OF COMORBIDITY**		**ELIXHAUSER COMORBIDITY MEASURE**	
**Geriatric Syndromes**	**OR (CI 95%)**	p^*^	**OR (CI 95%)**	p^*^	**OR (CI 95%)**	p^*^	**OR (CI 95%)**	p^*^
**Dementia**								
**Unadjusted**	1.311 (1.154–1.490)	< 0.01^*^	1.081 (0.970–1.205)	0.15	0.617 (0.429–0.888)	< 0.01^*^	1.166 (1.115–1.220)	< .001^*^
**Adjusted**^a^	1.295 (1.135–1.478)	< 0.01^*^	1.086 (0.971–1.214)	0.14	0.589 (0.404–0.859)	< 0.01^*^	1.158 (1.117–1.212)	< .001^*^
**Malnutrition**								
**Unadjusted**	1.152 (0.937–1.416)	0.17	0.942 (0.777–1.142)	0.54	1.702 (0.970–2.987)	0.06	1.144 (1.074–1.219)	< .001^*^
**Adjusted**^a^	1.117 (0.907–1.377)	0.29	0.926 (0.756–1.133)	0.454	1.572 (0.893–2.765)	0.117	1.134 (1.063–1.210)	< .001^*^
**Slow walking speed**								
**Unadjusted**	1.132 (1.001–1.280)	0.04^*^	1.149 (1.033–1.278)	0.01^*^	1.312 (0.951–1.810)	0.09	1.003 (0.971–1.036)	0.85
**Adjusted**^a^	1.230 (1.056–1.433)	< 0.01^*^	1.062 (0.936–1.204)	0.02	1.445 (0.941–2.220)	0.09	1.068 (1.020–1.117)	0.74
**Depression**								
**Unadjusted**	1.015 (0.907–1.135)	0.79	0.972 (0.883–1.071)	0.56	1.003 (0.743–1.353)	0.98	0.960 (0.929–0.992)	0.01^*^
**Adjusted**^a^	1.022 (0.911–1.146)	0.71	0.965 (0.874–1.065)	0.47	0.970 (0.715–1.317)	0.84	0.962 (0.930–0.995)	0.02^*^
**Polypharmacy**								
**Unadjusted**	1.925 (1.616–2.295)	< .001^*^	2.668 (2.153–3.306)	< .001^*^	2.502 (1.737–3.604)	< .001^*^	1.088 (1.048–1.130)	< .001^*^
**Adjusted**^a^	1.917 (1.604–2.289)	< .001^*^	2.700 (2.173–3.355)	< .001^*^	2.540 (1.755–3.677)	< .001^*^	1.084 (1.043–1.126)	< .001^*^
**Frailty**								
**Unadjusted**	1.250 (1.086–1.439)	< .001^*^	1.149 (1.020–1.294)	0.02^*^	1.535 (1.040–2.267)	0.03^*^	1.113 (1.063–1.164)	< .001^*^
**Adjusted**^a^	1.221 (1.052–1.416)	< 0.01^*^	1.117 (0.985–1.266)	0.08	1.453 (0.952–2.216)	0.08	1.119 (1.066–1.174)	< .001^*^
**Urinary Incontinence**								
**Unadjusted**	1.119 (0.999–1.253)	0.05	1.061 (0.964–1.167)	0.22	1.188 (0.880–1.603)	0.26	1.028 (0.995–1.062)	0.09
**Adjusted**^a^	1.091 (0.972–1.225)	0.13	1.050 (0.953–1.157)	0.32	1.140 (0.840–1.548)	0.40	1.021 (0.987–1.055)	0.22
**Falls**								
**Unadjusted**	1.074 (0.960–1.201)	0.21	1.051 (0.955–1.157)	0.31	1.366 (1.008–1.851)	0.04^*^	1.003 (0.971–1.036)	0.85
**Adjusted**^a^	1.048 (0.933–1.176)	0.42	1.049 (0.951–1.157)	0.33	1.370 (1.002–1.873)	0.04^*^	0.993 (0.961–1.027)	0.68
**Orthostatic Hypotension**								
**Unadjusted**	1.129 (0.976–1.306)	0.10	1.076 (0.947–1.223)	0.26	1.276 (0.851–1.915)	0.23	1.036 (0.993–1.080)	0.10
**Adjusted**^a^	1.111 (0.953–1.296)	0.17	1.079 (0.947–1.230)	0.25	1.370 (0.898–2.089)	0.14	1.026 (0.982–1.072)	0.25
**Low muscle strength**								
**Unadjusted**	1.200 (1.031–1.397)	0.01^*^	1.137 (1.000–1.292)	0.05	1.156 (0.774–1.725)	0.47	1.018 (0.976–1.062)	0.39
**Adjusted**^a^	1.192 (1.013–1.402)	0.03^*^	1.101 (0.964–1.258)	0.15	1.044 (0.680–1.603)	0.84	1.018 (0.973–1.065)	0.44

^a^Adjustments were made according to age and sex

^*^Bold p values indicate statistical significance *p* < 0.05

According to the reference scores, multinomial regression analyses with CCI, ECM, and GIC subgroups showed no linear relationship between indices and MMSE, YGDS, POMA, MNA ADLs, Fried frailty score, number of drugs, LOS. Also, the Group 4 of ECM subgroups was associated with MMSE, MNA, ADLs, frailty scores, and total drug number as well as the LOS, and the Group 4 of CCI subgroups was associated with Basic ADLs, frailty score, the number of drugs, and LOS, the Group 4 of GIC was related to the only number of drugs compared to the reference groups when age and gender-adjusted (Table 3).

**Table 3:** Multinomial regression analyses of indices’ subgroups

	MMSE		YGDS		POMA_T_		Timed Up&Go		MNA-SF		BADL		IADL		Frailty score		Number of drugs		LOS	
	**OR** **(CI 95%)**	**p**	**OR** **(CI 95%)**	**p**	**OR** **(CI 95%)**	**p**	**OR** **(CI 95%)**	**p**	**OR** **(CI 95%)**	**p**	**OR** **(CI 95%)**	**p**	**OR** **(CI 95%)**	**p**	**OR** **(CI 95%)**	**p**	**OR** **(CI 95%)**	**p**	**OR** **(CI 95%)**	**p**
**Elixhauser Comorbidity Index**																				
**Group 1**	**Reference group**																			
**Group 2**	0.93 (0.86–1.02)	0.14	0.89 (0.75–1.04)	0.16	1.01 (0.95–1.07)	0.62	0.96 (0.91–1.01)	0.14	0.92 (0.71–1.20)	0.57	1.00 (0.97–1.03)	0.80	0.99 (0.95–1.03)	0.73	0.78 (0.52–1.16)	0.23	0,98 (0.86–1.11)	0.78	1.18 (1.00–1.38)	**0.04**
**Group 3**	1.01 (0.92–1.10)	0.81	1.02 (0.92–1.14)	0.59	1.00 (0.95–1.05)	0.93	0.98 (0.95–1.01)	0.33	0.77 (0.62–0.94)	***0.01***	0.99 (0.96–1.01)	0.46	0.99 (0.95–1.02)	0.60	0.95 (0.68–1.31)	0.76	0.99 (0.89–1.10)	0.92	1.01 (0.86–1.18)	0.88
**Group 4**	0.88 (0.83–0.93)	** < 0.01**	1.05 (0.96–1.14)	0.23	0.97 (0.93–1.01)	0.16	1.00 (0.98–1.02)	0.95	0.63 (0.53–0.76)	** < 0.01**	0.97 (0.95–0.99)	** < 0.01**	0.95 (0.92–0.99)	**0.01**	1.42 (1.11–1.80)	** < 0.01**	1.12 (1.03–1.21)	** < 0.01**	1.11 (0.99–1.25)	**0.04**
**Charlson Comorbidity Index**																				
**Group 1**	**Reference group**																			
**Group 2**	0.94 (0.89–1.01)	0.10	0.94 (0.85–1.04)	0.25	0.99 (0.95–1.03)	0.71	1.03 (0.99–1.07)	0.12	0.97 (0.84–1.12)	0.72	0.99 (0.97–1.01)	0.52	0.99 (0.97–1.02)	0.91	1.15 (0.86–1.53)	0.33	1.30 (1.14–1.48)	** < 0.01**	1.06 (0.92–1.21)	0.37
**Group 3**	0.96 (0.89–1.02)	0.22	1.08 (0.98–1.20)	0.10	0.98 (0.93–1.02)	0.38	1.04 (1.00–1.08)	**0.03**	0.89 (0.77–1.03)	0.14	0.97 (0.95–1.00)	0.06	0.95 (0.91–0.99)	**0.02**	1.72 (1.26–2.36)	** < 0.01**	1.66 (1.43–1.92)	** < 0.01**	1.12 (0.97–1.29)	0.10
**Group 4**	0.97 (0.90–1.04)	0.46	0.97 (0.86–1.10)	0.70	0.98 (0.93–1.03)	0.57	1.03 (0.99–1.08)	0.07	0.92 (0.78–1.08)	0.32	0.97 (0.94–0.99)	**0.01**	0.95 (0.92–1.00)	0.09	1.45 (1.02–2.06)	**0.03**	1.77 (1.52–2.07)	** < 0.01**	1.18 (1.01–1.38)	**0.032**
**Geriatric Index of Comorbidity**																				
**Group 1**	**Reference group**																			
**Group 2**	1.00 (0.95–1.06)	0.75	1.02 (0.90–1.15)	0.73	0.99 (0.95–1.03)	0.70	1.01 (0.98–1.05)	0.27	1.02 (0.89–1.17)	0.74	0.99 (0.97–1.01)	0.61	0.99 (0.96–1.02)	0.62	1.19 (0.88–1.61)	0.25	1.36 (1.20–1.54)	** < 0.01**	1.00 (0.87–1.15)	0.95
**Group 3**	1.00 (0.94–1.06)	0.91	1.08 (0.96–1.22)	0.17	0.94 (0.88–1.00)	0.06	1.02 (0.99–1.06)	0.09	0.91 (0.80–1.04)	0.18	0.98 (0.96–1.00)	0.25	0.96 (0.92–1.01)	0.17	1.26 (0.91–1.72)	0.15	1.42 (1.23–1.64)	** < 0.01**	1.13 (0.99–1.28)	0.05
**Group 4**	1.14 (0.98–1.33)	0.76	1.25 (0.89–1.74)	0.18	0.93 (0.85–1.01)	0.09	1.03 (0.97–1.09)	0.29	0.82 (0.65–1.03)	0.08	0.98 (0.95–1.01)	0.25	0.96 (0.87–1.06)	0.45	1.79 (0.68–4.73)	0.23	1.47 (1.11–1.95)	** < 0.01**	1.33 (0.98–1.79)	0.06

*BADL* Basic Activities of Daily Living, *CCI* Charlson Comorbidity Index, *GIC* Geriatric Index of Comorbidity, *IADL* Instrumental Activities of Daily Living, *ECM* Elixhauser Comorbidity Index, *LOS* Length of stay, *MMSE* Mini-Mental State Examination, *MNA-SF* Mini-Nutritional Assessment-Short Form, *POMA*_T_ Performance-oriented Mobility Assessment-Total, *YGDS* Yesevage Geriatric Depression

## Discussion

This cross-sectional study suggests that none of these four comorbidity indices, which are expected to represent the older adults as a whole, fully complied with CGA or geriatric syndromes. However, it was also demonstrated that CCI and ECM were related to frailty, dementia, polypharmacy, whereas ECM was also associated with malnutrition. Moreover, although the ECM subgroup 4 seems to be closely associated with ADL, MMSE, nutritional assessment scores, and LOS, there was no linear relationship between the subgroups.

Considering that 92% of older adults have at least one chronic disease or geriatric syndrome, and at least 30% use five or more medications, it is better to understand how heterogeneous these patients are. [26, 31] Comorbidities and geriatric syndromes commonly overlap, and cause healthcare needs more. [32] However, the coexistence of different comorbidities may not always represent the clinical complexity of the older patient. Additional health conditions such as sensory deficits, mobility problems, nutritional deficiency should be considered as a holistic approach. Therefore, a comorbidity index that can reflect this heterogeneity and be compatible with CGA is of great importance for geriatric practice. [11, 13] CCI, ECM, GIC, and MCI have commonly used four medical practice indices, of which CCI is the most widely used one to have prognostic value on mortality. [33–37] ECM is another frequently used index reported to predict mortality and LOS. [38–42] Another index, MCI, is a drug-based index that primarily focuses on polypharmacy. [30] The other index, as different from others, GIC, has been developed for geriatric patients to evaluate the disease severity. [11] The most common criticism of available comorbidity indices is their inability to reflect functional and cognitive capacity in older adults. [43] Most of the studies, thus far, on comorbidity indices have aimed to predict the in-hospital or post-discharge mortality of the patients, which are too insufficient to meet the needs of the geriatric practice. Also, as far as we are concerned, the relationship between CGA and geriatric syndromes and comorbidity indices has not been studied yet.

This study found that CCI was correlated with LOS and most CGA parameters, such as physical fitness, nutritional status, functionality, frailty, and number of drugs, in older patients. Consistent with our results, many studies have shown that CCI is associated with ADLs and functionality. [44–46] In contrast to our result, a study in older patients from Singapore reported that CCI was not associated with LOS, which was more than 21 days. [30] However, since LOS longer than 21 days is quite a long time for general geriatric practice, this result cannot be mentioned to reflect daily geriatric practice. In another study, including long-term care facility residents with about half of dementia, a negative correlation was reported between CCI and MMSE. [47] Given that approximately half of the patients were demented in that study, [47] this result is not surprising and cannot reflect the geriatric population. Furthermore, although there was no relation between MMSE and CCI scores, CCI was related to dementia in the present study. Consequently, regarding the association of CCI with geriatric syndromes, depressive mood and nutritional status should be considered as shortcomings of CCI, and increasing the index's compatibility with the CGA will enable it to be used more effectively and widely.

Previous studies reported that ECM predicted LOS better than CCI in the general population, [38, 48, 49] and the van Walraven adaptation of the ECM was also developed to predict mortality. [29] Among these four indices, ECM was the only index associated with MMSE. Additionally, the ECM subgroup 4 was the most related group to CGA parameters. Thus, to the best of our knowledge, this is the first study to compare ECM with CGA and, once its shortcomings are eliminated, ECM will be a handy scale for geriatric practice.

Given the importance and complexity of pharmacotherapy in geriatric cases and polypharmacy, a geriatric syndrome, [13] it would be rational for drugs to be included in the geriatric comorbidity indices. Following this, we showed that MCI, a drug-based comorbidity index, was most closely associated with polypharmacy as expected. Additionally, it reflects frailty and walking speed. However, nutritional status, major neurocognitive disorder, and depressive mood may be overlooked by the MCI. Therefore, MCI is a relatively new scale and needs further studies.

The GIC, the last scale we evaluated, was developed especially for geriatric cases; however, we found that it was not associated with nutritional status, cognitive status, and depressive mood. Moreover, GIC was inversely related to dementia. Accordingly, Rozzini et al. also reported that GIC was correlated with ADL, not IADL, using the Katz index as in our study. [11] This may be because although the index was developed for older patients, it did not focus mainly on geriatric syndromes or AGD parameters.

Frailty and polypharmacy were both related to the four indices. GIC had higher odds for both geriatric syndromes. The relationship may be explained index characteristics for assessing the severity of the diseases. Besides, ECM and CCI had a closer relationship with CGA parameters. In clinical practice, patients with higher scores of GIC, ECM, or CCI should be part of a further evaluation to provide a comprehensive management plan.

On the other hand, no linear relationship was observed when the CCI, GIC, and ECM groups were categorized according to increasing comorbidity scores. The low number of patients may explain it in the subgroups and the heterogeneity of patients. Moreover, considering depression is a common geriatric syndrome, it is remarkable that it was not associated with the depressive mood at four indices. Furthermore, ECM was inversely related to depression. Therefore, it is essential to evaluate depressive mood in studies to increase the compatibility of these scales with CGA and develop new comorbidity scales for older adults. [50]

Our study's strengths are an adequate sample size, such as 366 geriatric cases, and performing CGA to each patient. In addition, this study is one of the first studies evaluating the relationship between four comorbidity scales and geriatric syndromes. The limitations of our research are retrospective and cross-sectional design and the inability to assess mortality.

## Conclusions

The present comorbidity scales are insufficient to reflect geriatric syndromes simultaneously. New indices should be developed considering the complexity of the older geriatric patients and the limitations of the existing ones.

## Data Availability

The datasets generated and/or analyzed during the current study are not the publicly available due presence of private information but are available from the corresponding author on reasonable request.

## Abbreviations

CCI: Charlson Comorbidity Index
ECM: Elixhauser Comorbidity Index
GIC: Geriatric Index of Comorbidity
MCI: Medicine Comorbidity Index
CGA: Comprehensive Geriatric Assessment
BADL: Basic Activities of Daily Living
IADL: Instrumental Activities of Daily Living
BMI: Body Mass Index
LOS: Length of stay
MMSE: Mini-Mental State Examination
MNA-SF: Mini-Nutritional Assessment-Short Form
OH: Orthostatic Hypotension
POMA: Performance-oriented Mobility Assessment
YGDS: Yesevage Geriatric Depression Scale
